# Retrieval of Migrated Coils From Distal Cerebral Vasculature Using Stent Retriever: A Case Series

**DOI:** 10.7759/cureus.37213

**Published:** 2023-04-06

**Authors:** Ashish Kulhari, Farah Fourcand, Amrinder Singh, Haralabos Zacharatos, Siddhart Mehta, Jawad F Kirmani

**Affiliations:** 1 Department of Neurology, Research Medical Center, Kansas City, USA; 2 Department of Neurology, University of Missouri Kansas City School of Medicine, Kansas City, USA; 3 Department of Medicine, Kansas City University of Medicine and Biosciences, Kansas City, USA; 4 Department of Neurology, Hackensack Meridian JFK University Medical Center, Edison, USA; 5 Department of Neurology, United Health Services (UHS) Binghamton General Hospital, Johnson City, USA

**Keywords:** coil herniation, transarterial coil embolization, stent retriever, coil migration, intracranial aneurysm

## Abstract

The incidence of coil dislocation during an endovascular embolization of intracranial aneurysm is low but it can lead to serious thrombo-embolic complications. Therefore, coil displacement/migration often requires either retrieval or fixation of the errant coil with a stent. There are no standard recommended methods of coil retrieval. We present a series of three cases in which off-label application of a stent retriever allowed successful retrieval of herniated coils.

## Introduction

Endovascular coil embolization of intracranial aneurysms has become a widely accepted treatment, especially for ruptured aneurysms, because of its less invasive nature and lower rates of complications [1-3]. Intra-procedure aneurysm rupture and thromboembolic complications remain the greatest risk in the endovascular treatment of an aneurysm [4]. Coil dislocation/migration contributes significantly to thromboembolic complications [4-6]. Multiple devices including Amplatz microsnare (Ev3, Irvine, CA, USA) [7], Retriever microcatheter (Target Therapeutics, Fremont, CA, USA) [8], Alligator Retrieval Device (Chestnut Medical Technologies, Menlo Park, CA, USA) [9], MERCI (Concentric Medical, Mountain View, CA, USA) [10] and Stent retrievers [4,6,11-16] have been described for retrieval of dislocated coils.

Based on the successful results of multiple large randomized controlled trials, stent retrievers are used worldwide for thrombus retrieval in intracranial large vessel occlusions [17-21]. Given the very limited evidence, consensus does not exist regarding the best retrieval technique for migrated coils. We report our series of three patients who underwent successful retrieval of displaced coils using a stent retriever, to strengthen the evidence on the feasibility, safety, and efficacy of coil retrieval using a stent retriever.

## Case presentation

Case 1

**Figure 1:**
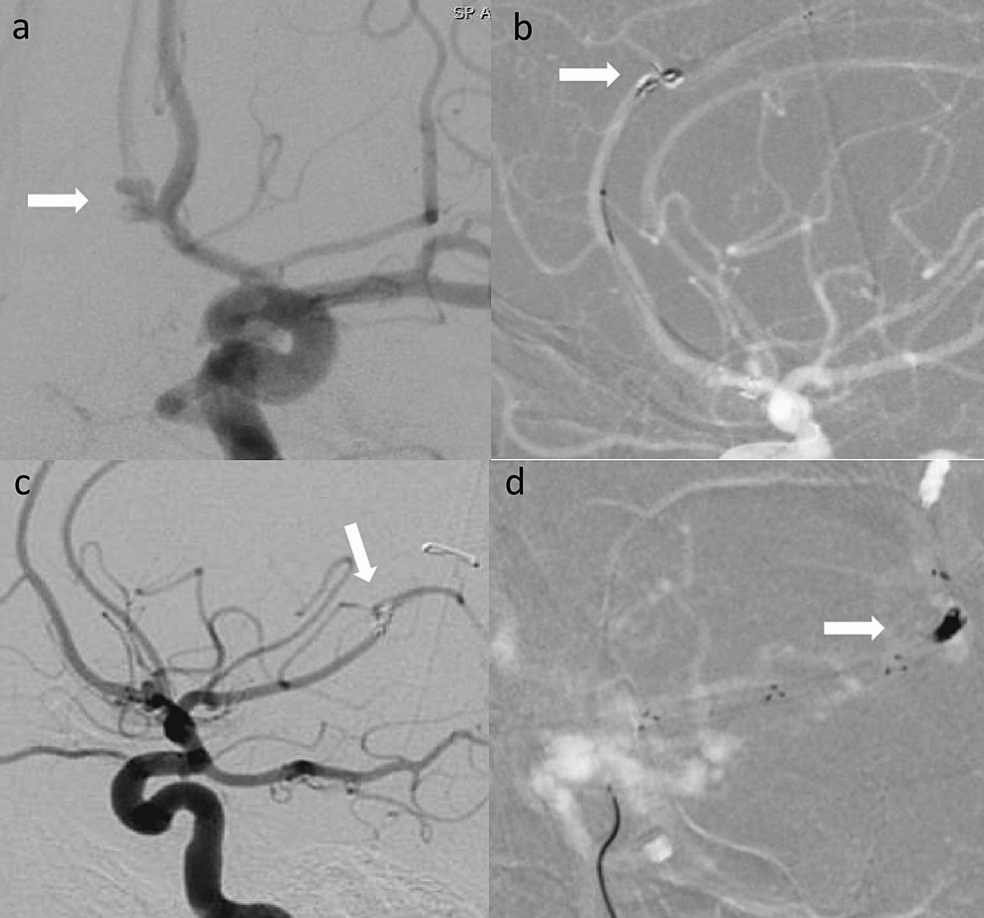
(a) Left ICA injection (AP view) shows an irregular, wide necked, trilobed anterior communicating artery aneurysm. (b) Left ICA road map (lateral view) shows stent retriever deployed across the herniated coil mass in left ACA. (c) Left ICA injection (lateral view) shows herniated coil mass in the M2 division of left MCA. (d) Left ICA road map (lateral view) shows stent retriever deployed across the herniated coil mass in left MCA. ICA: internal carotid artery; AP: anterior-posterior; ACA: anterior cerebral artery; MCA: middle cerebral artery.

56-year-old woman with a history of hypertension and cigarette smoking was admitted with Hunt & Hess 2, modified Fischer 4, aneurysmal subarachnoid hemorrhage. Biplane digital subtraction angiography (DSA) revealed an irregular trilobed 4mm x 3mm wide neck anterior communicating artery (ACOM) aneurysm (Figure 1a). Three Galaxy orbit complex xtrasoft 2mm x 2cm coils (Codman Neurovascular, Raynham, MA, USA) were deployed through SL 10 microcatheter (Stryker Neurovascular, Fremont, CA, USA) to embolize the aneurysm. During the placement of the third coil, the first two coils dislodged from the aneurysm and migrated into the A2 segment of the left anterior cerebral artery (ACA). Eptifibatide bolus was administered immediately. Coil retrieval with a stent retriever was decided. 8F Flowgate balloon guide catheter (Stryker Neurovascular, Fremont, CA, USA) was parked in the left ICA bulb. 5F Sophia intermediate catheter (Microvention, Aliso Viejo, CA, USA), and Velocity microcatheter (Penumbra, Alameda, CA, USA) were navigated intracranially over the Transcend 014 microwire (Boston Scientific, Marlborough, MA, USA). Sophia was parked at the carotid terminus. The coil mass was crossed with microwire and microcatheter was carefully advanced distal to it. Trevo 3 x20 stent retriever (Stryker Neurovascular, Fremont, CA, USA) was deployed across the coil mass (Figure 1b). Velocity was withdrawn from the body. Using the TRAP technique, stent retriever was dragged back slowly while maintaining suction through Sophia and proximal flow arrest through Flowgate. At the carotid terminus, coil mass got dislodged from stent retriever and migrated to M2 division of left middle cerebral artery (MCA) (Figure 1c). Solitaire 4 x 40 (Medtronic, Minneapolis, MN, USA) stent retriever was used successfully to retrieve the coil mass from left MCA using similar technique as described above (Figure 1d). 

Case 2

**Figure 2:**
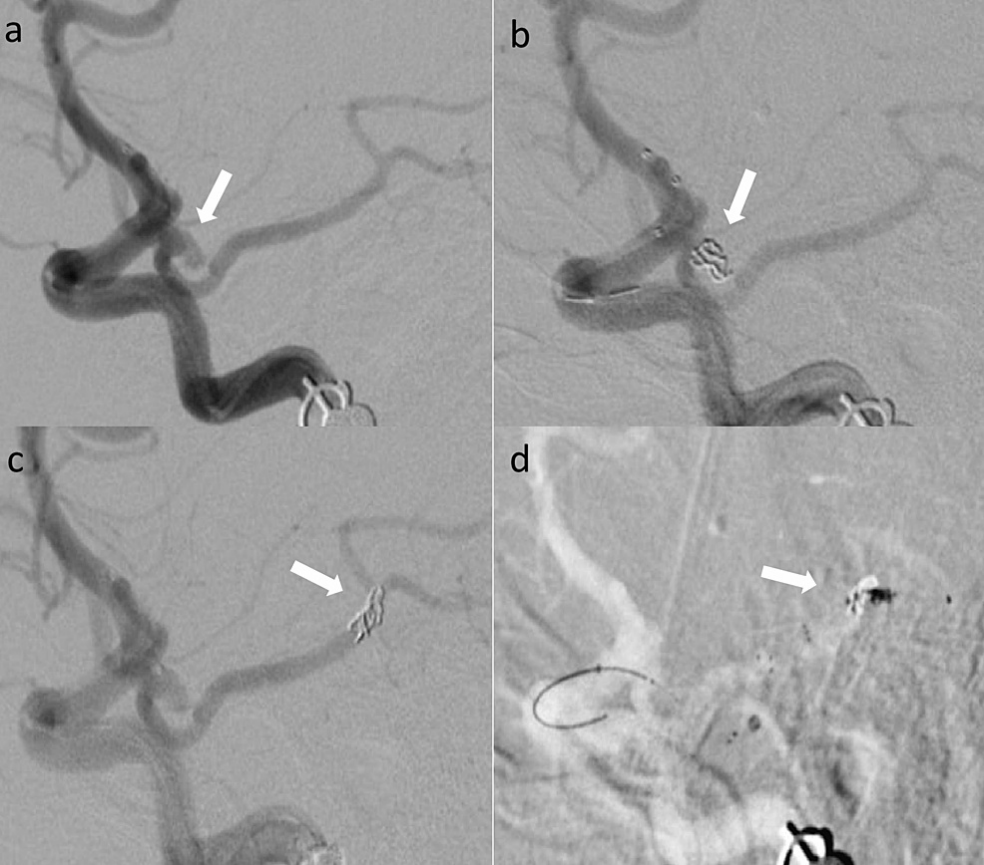
(a) Left ICA injection (lateral view) shows an irregular, wide necked, posterior communicating artery aneurysm. (b) Left ICA injection (lateral view) shows the deployed coil mass within the aneurysm. (c) Left ICA injection (lateral view) shows herniated coil mass in the left PCA. (d) Left ICA road map (lateral view) shows stent retriever deployed across the herniated coil mass. ICA: internal carotid artery; PCA: posterior cerebral artery.

A 64-year-old woman with history of hypertension was admitted with Hunt & Hess 5, modified Fischer 4, aneurysmal subarachnoid hemorrhage. Biplane DSA revealed an irregular 5.3mm x 3mm wide neck left posterior communicating artery (PCOM) aneurysm with fetal left posterior cerebral artery (PCA) coming off the neck of aneurysm (Figure 2a). One Galaxy orbit complex xtrasoft 2.5mm x 3.5cm coil (Codman Neurovascular, Raynham, MA, USA) was deployed through the SL 10 microcatheter (Stryker Neurovascular, Fremont, CA, USA) to embolize the aneurysm (Figure 2b). Follow up angiogram showed dislocated coil mass in left PCA (P2-P3 junction) (Figure 2c). Eptifibatide bolus was administered immediately. Similar to previous case, Flowgate (Stryker Neurovascular, Fremont, CA, USA) was parked in the left ICA bulb. 3MAX intermediate catheter (Penumbra, Alameda, CA, USA), Velocity microcatheter (Penumbra, Alameda, CA, USA) were navigated intracranially over the Synchro 014 (Stryker Neurovascular, Fremont, CA, USA) microwire. 3MAX was parked in the posterior communicating artery. Coil mass was carefully crossed with microwire and microcatheter was advanced distal to the coil mass. Solitaire 4 x 40 stent retriever (Medtronic, Minneapolis, MN, USA) was deployed across the coil mass (Figure 2d). Using the TRAP technique, stent retriever was dragged back slowly while maintaining suction through 3MAX and flow arrest through Flowgate. Coil mass was successfully retrieved after two attempts.

Case 3

**Figure 3:**
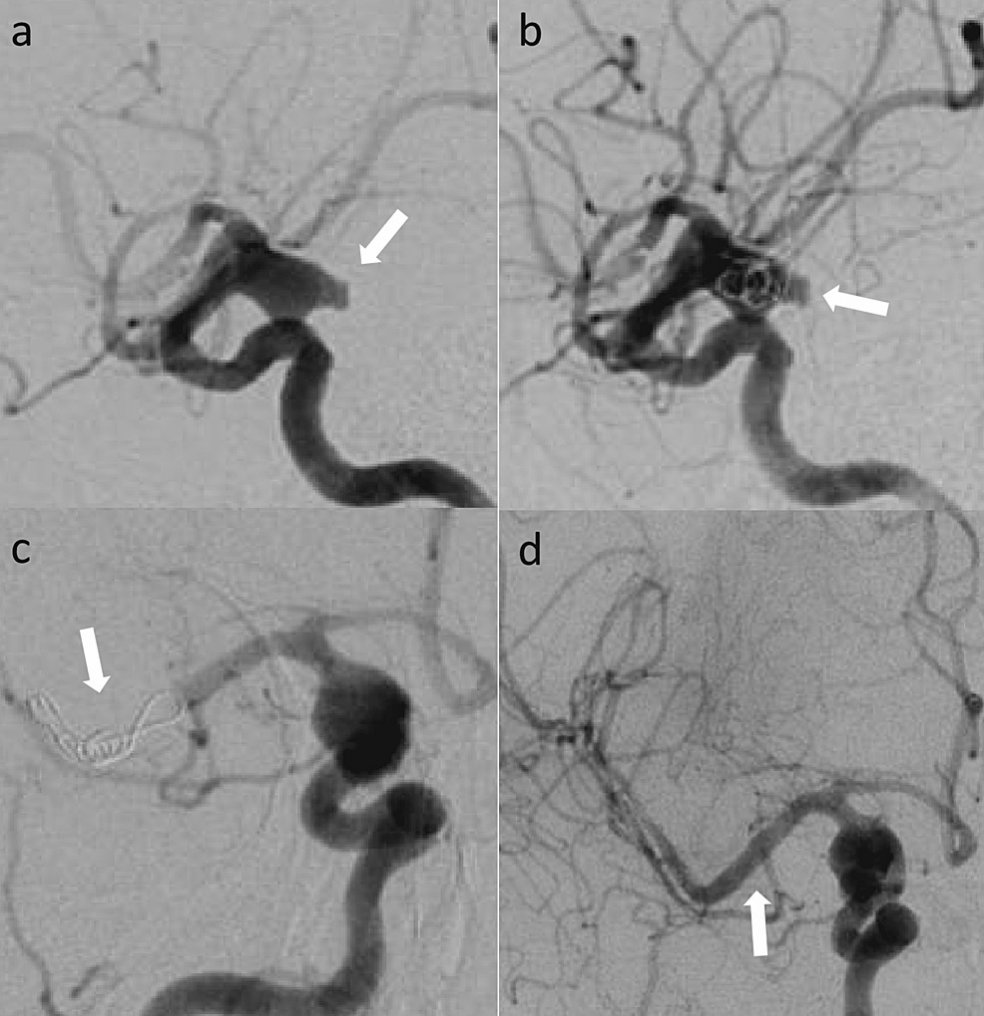
(a) Right ICA injection (lateral view) shows an irregular, wide necked, bilobed supraclinoid ICA aneurysm. (b) Right ICA injection (lateral view) shows deployed coil mass within the aneurysm. (c) Right ICA injection (AP view) shows herniated coil mass in the right MCA bifurcation with occlusion of right MCA. (d) Right ICA injection (AP view) shows recanalization of right MCA post retrieval of coil mass with stent retriever. ICA: internal carotid artery; AP: anterior-posterior; MCA: middle cerebral artery.

A 65-year-old woman with history of hypertension was admitted with Hunt & Hess 2, modified Fischer 4, aneurysmal subarachnoid hemorrhage. Biplane DSA revealed an irregular bilobed 8.5mm x 6.8mm wide neck right supraclinoid ICA aneurysm (Figure 3a). One Galaxy orbit complex xtrasoft 5mm x 10cm coil (Codman Neurovascular, Raynham, MA, USA) was deployed through the SL 10 microcatheter (Stryker Neurovascular, Fremont, CA, USA) to embolize the aneurysm (Figure 3b). Follow up angiogram showed dislocated coil mass in the distal M1 segment of right MCA (Figure 3c). Similar technique, as described in previous cases, was used to retrieve the dislocated coil using stent retriever. Figure 3d shows recanalization of right MCA post retrieval of coil mass.

Table 1 summarizes the patient demographics, aneurysm features and endovascular tools used for coil retrieval in the three cases.

**Table 1:** patient demographics, aneurysm features and endovascular tools used for coil retrieval in the three consecutive cases. F: female; HTN: hypertension; HH: Hunt & Hess score; MFS: Modified Fischer score; ACOM: Anterior communicating artery; PCOM: Posterior communicating artery; ICA: Internal carotid artery; ACA: Anterior cerebral artery; MCA: Middle cerebral artery; PCA: Posterior cerebral artery

Case	Age	Sex	Risk factors	HH	MFS	Aneurysm features	Coils	Vessel of coil migration	Eptifibatide	Stent retriever	No of attempts	Complications
1	56	F	HTN, smoker	2	4	Irregular, trilobed, 4mm x 3mm, wide neck, ACOM aneurysm	Three Galaxy Orbit complex xtrasoft coils (2mm x 2cm)	Left ACA (A2-A3 junction) Left MCA (M2 branch)	Yes	Trevo 3x20; Solitaire 4x40	2	None
2	64	F	HTN	5	4	Irregular, 5.3mm x 3mm, wide neck, left PCOM aneurysm	One Galaxy Orbit complex xtrasoft coil (2.5mm x 3.5cm)	Left PCA (P2-P3 junction)	Yes	Solitaire 4x40	2	Mild vasospasm
3	65	F	HTN	2	4	Irregular, bilobed, 8.5mm x 6.8mm, wide neck, right supraclinoid ICA aneurysm	One Galaxy Orbit complex xtrasoft coil (5mm x 10cm)	Right MCA (distal M1 segment)	Yes	Solitaire 4x40; Trevo 3x20	2	Mild vasospasm

## Discussion

Endovascular coil embolization of intracranial aneurysms has become a widely accepted treatment, especially for ruptured aneurysms, because of its less invasive nature and significantly lower rates of complications [1-3]. The incidence of periprocedural mortality and severe disability with endovascular coil embolization is around 2%-6% [3,22-25]. Intra-procedure aneurysm rupture and thromboembolic complications remain the greatest risk in the endovascular treatment of an aneurysm [4]. Coil dislocation/migration contributes significantly to thromboembolic complications [4-6]. Although coil displacement typically occurs during the placement of the finishing coil, it can also occur with initial coil placement specially in small aneurysms [26]. The risk for coil dislocation and migration depends on aneurysm anatomy including size, shape, neck width, dome:neck ratio, aspect ratio, location of the aneurysm, intra-aneurysm blood flow dynamics post-coil embolization, and technical factors like coil size, microcatheter stability, etc. [6,26,27]. Small irregular wide neck (>4mm) aneurysms with low aspect and dome:neck ratios have higher chances of coil dislocation/migration. Using a balloon or stent along with careful coil deployment while watching the microcatheter position can minimize the risk of coil displacement [6,27].

Coil dislocation may range from minimal protrusion of single loops of the coil into the parent vessel to migration of the coil into a distal blood vessel. Asymptomatic single loops of herniated coils are best treated conservatively with a short course of intravenous glycoprotein IIb/IIIa antagonists or anticoagulation followed by antiplatelet therapy that prevents potential thrombus formation until endothelialization of the coil loops makes them less thrombogenic [6,26]. However complete coil mass dislocation and migration can cause large vessel occlusion leading to large infarct and death. When such severe consequences are anticipated, coil dislocation does require intervention [13,26]. Interventions for dislocated coils are broadly divided into the fixation of the dislocated coil along the vessel wall using an intracranial stent or retrieval of the displaced coil mass. Although stent placement across the errant coil mass is a very efficient and safe technique, concern about placing another thrombogenic implant and the need for dual antiplatelet therapy makes it less attractive to most the neurointerventionalists, especially in ruptured aneurysms [6,26,28]. Hence, retrieval of the dislocated coil is often the primary salvage technique.

Multiple devices have been described previously for retrieval of dislocated coils. Watanbe et al. successfully retrieved a migrated coil from the superior cerebellar artery using the Retriever 18 microcatheter (Target Therapeutics, Fremont, CA, USA) [8]. Henkes et al. used an Alligator Retrieval Device (Chestnut Medical Technologies, Menlo Park, CA, USA) for successful endovascular removal of foreign bodies from intracranial arteries [9]. Dinc et al. reported the successful use of Amplatz Gooseneck Microsnare (Ev3, Irvine, CA, USA) in two patients [7]. Vora et al. described a case of successful retrieval of the dislocated coil with Merci Retrievers (Concentric Medical, Mountain View, CA, USA) [10]. Lee et al. described a simple technique using a wire as a snare for the removal of displaced and stretched coils [29].

Wakhloo and Gounis were the first to demonstrate the use of a retrievable closed-cell intracranial stent to successfully retrieve coil mass in an aneurysm model [30]. O´Hare et al. described the first successful human case of dislocated coil retrieval using a Solitaire stent retriever (Medtronic, Minneapolis, MN, USA) in 2010 [11]. To the best of our knowledge, there is a total of 2 case series and 6 case reports published in the literature so far on coil retrieval using a stent retriever [4,6,11-16]. Given the very limited evidence, consensus does not exist regarding the best retrieval technique for migrated coils. With the recent increase in stent retriever usage, given their well-documented superiority in treating acute ischemic stroke due to emergent large vessel occlusions, neuro interventionalist across the world feel much more comfortable using these devices to extract migrated coils [17-21]. The superiority of stent retrievers over traditional retrieval devices for coil retrieval is due to multiple points of contact and engagement with the target thrombus. In addition, stent retrievers retain their structural integrity compared with traditional retrieval devices, which can unwind and slide through the target, thus causing a loss of target engagement during the pull [13]. Another advantage of using a stent retriever over a traditional retrieval device is the immediate restoration of blood flow to distal vessels once the stent is deployed.

Given the paucity of literature on the usage of a stent retriever for coil retrieval, we report our series of three patients that underwent successful retrieval of displaced coils using a stent retriever to strengthen the evidence on feasibility, safety, and efficacy of coil retrieval using stent retriever.

## Conclusions

Based on previously published case reports/series and our experience, we conclude stent retrievers, although not approved for retrieval of dislocated coils, are safe and effective in dislocated coil retrieval during aneurysm coiling. Given the extensive usage of stent retrievers for mechanical thrombectomy in large vessel occlusions, neurointerventionalists worldwide would feel more comfortable using stent retrievers over the older retrieval devices for retrieval of migrated coils.
